# Sensor-Based Real-Time Detection in Vulcanization Control Using Machine Learning and Pattern Clustering

**DOI:** 10.3390/s18093123

**Published:** 2018-09-16

**Authors:** Jonghyuk Kim, Hyunwoo Hwangbo

**Affiliations:** 1 Department of Global Economics, Gachon University, Gyeonggi-do 13120, Korea; halfmoonlike@gmail.com; 2 Department of Data & Knowledge Service Engineering, Dankook University, Gyeonggi-do 16890, Korea

**Keywords:** synthetic rubber compounds, vulcanization process, sensor-based real-time detection model, pattern similarity cluster

## Abstract

Recent paradigm shifts in manufacturing have resulted from the need for a smart manufacturing environment. In this study, we developed a model to detect anomalous signs in advance and embedded it in an existing programmable logic controller system. For this, we investigated the innovation process for smart manufacturing in the domain of synthetic rubber and its vulcanization process, as well as a real-time sensing technology. The results indicate that only analysis of the pattern of input variables can lead to significant results without the generation of target variables through manual testing of chemical properties. We have also made a practical contribution to the realization of a smart manufacturing environment by building cloud-based infrastructure and models for the pre-detection of defects.

## 1. Introduction

Signs of manufacturing reshoring are being detected in manufacturing upbringing policies worldwide. In particular, since the 2008 financial crisis, the strategy of creating value through manufacturing in the low-growth global economic environment is proceeding very rapidly under the leadership of manufacturing-led countries. In addition to Germany’s Industry 4.0, the US Advanced Manufacturing Partnership and Japan’s Industrial Reconstruction Plan are emerging as major national projects [1]. This strategy originates from the belief that when the manufacturing industry is integrated with smart technology, including information and communications technology (ICT), a new type of industry can be created that has a very large ripple effect. In particular, measurement science for additive manufacturing, which leads the manufacturing industry in the US, aims to develop automation models for tasks such as material characterization, sensor measurement and monitoring, and database preparation through performance verification and digitization [1,2]. This paradigm shift in manufacturing has resulted from changes in the smart manufacturing environment. We compare the paradigm differences between the existing manufacturing environment and the smart manufacturing environment by major criteria. Currently, more manufacturing-based servitized industrial companies are being established compared to pure manufacturing companies. Data collection and utilization, or decision cycle, has been changed to real-time big data instead of periodic sample data [3]. The smart manufacturing paradigm is a platform that allows all participants on the supply chain to converge equipment and information and to make rapid decisions in real time by integrating smart technology.

Synthetic rubber is mainly commercialized through molding and vulcanization processes. However, the current process used in the industry is not significantly advanced compared to the traditional process. In other words, it relies on laboratory-based chemical testing and field experts’ manufacturing expertise. In this study, we explore the innovation process for smart manufacturing in the industry that produces shoe soles, one of the synthetic rubber materials. Further, we improve the manufacturing process with sensor-based real-time control and build a failure control model with a smart manufacturing environment.

We first review previous studies on real-time sensing technology for synthetic rubber, vulcanization, and manufacturing. Second, we show how to construct big data infrastructure, a data preprocessing technique, and methodology for model building based on the vulcanization process data of company-A. Third, we present the results from some machine learning (ML) algorithms, including pattern similarity clusters, and explain their implications. Finally, we summarize the academic significance and practical contribution of this study, and identify directions for future research.

## 2. Background

### 2.1. Synthetic Rubber Compounds

We believe that consideration should be given to the industrial domain knowledge in order for this study to achieve its academic objectives more clearly. Domain knowledge includes a practical understanding of the industrial environment, terminology, and processes. To this end, we will introduce the classification and processing of synthetic rubber as follows. Synthetic rubber refers to plastic materials that can be chemical bonding substances or elastic substances having properties similar to those of natural rubber. The synthetic rubber industry mainly produces raw materials for tires, shoe soles, rubber hoses, wire clothing, conveyor belts, and other industrial rubber products [4]. Among various basic petrochemical materials, ethylene, propylene, and butadiene are the basic components extracted from naphtha and can be classified by heat separation and recovery. In this study, we will examine the types and uses of styrene butadiene rubber (SBR) and polybutadiene rubber (BR), which are typical synthetic rubber products extracted from butadiene and used in tires and rubber soles of shoes. As summarized in Table 1, SBR and BR are the most representative synthetic rubber products, accounting for more than 50% of the synthetic rubber demand worldwide [5,6].

**Figure 1:**
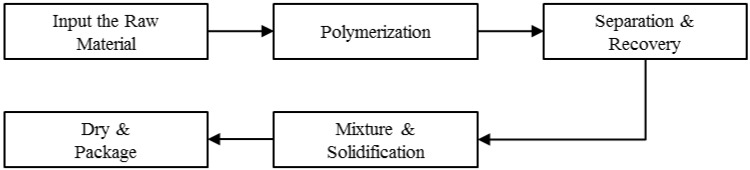
Flowchart of the styrene butadiene rubber (SBR) manufacturing process using emulsion polymerization.

Synthetic rubber materials are mainly manufactured using emulsion polymerization or solution polymerization. In particular, the manufacturing process of SBR, which uses the emulsion polymerization method, is the most widely used manufacturing method in the world. A flowchart of the manufacturing process of SBR is shown in Figure 1. First, butadiene and an emulsifier, which is a mixture of styrene and fatty acid, are fed into a reactor. Second, various chemicals are added to cause polymerization. Third, approximately 40% of unreacted raw materials are separated and recovered in the polymerization process. Fourth, latex from the recovery process is mixed with an antioxidant and an extended oil, following which it solidifies into small particles. Finally, the rubber, which contains moisture, is fully dried [7].

### 2.2. Curing Process (Vulcanization)

In order to determine the significance of our research, we think that it is important not only to gain a practical understanding of the synthetic rubber process, but also to summarize the theoretical background so far. Thus, we have summarized how academic discussions on synthetic rubber have been performed in recent years by dividing them into several categories. A curing process (or vulcanization) can be defined as a process in which a rubber material that has no elasticity is subjected to a chemical reaction by heat from the outside or by adding equivalent curatives and accelerators such as sulfur and peroxide, which have a specific performance (or elasticity). Vulcanization can cause significant differences in the performance or quality of the final product, depending on the control parameters such as heating temperature and time, the shape and size of the rubber material, and the accelerators. Many studies of the vulcanization process primarily focused on the temperature control and chemical control of the additives, which play the most important role in the rubber properties. We have focus on the research methods and the major implications of recent studies on the vulcanization process, as summarized in Table 2.

### 2.3. Sensor-Based Real-Time Detection in Manufacturing

As described in Table 2, automatic control technology employing real-time analysis in the real world is a very important factor in the technical and business aspects, in addition to the recent smart manufacturing technology. In particular, real-time automatic control technology is a means of strategic innovation in manufacturing through the fusion of human, machine, and information, and it is an exemplary combination of advanced ICT technology and manufacturing technology [25]. In order to keep pace with these trends, manufacturers have been working in recent years to develop key software and hardware for the cyber physical system (CPS), industrial Internet-of-things (IIoT), and real-time smart sensors [26]. They are also working to naturally apply these techniques in practice. Therefore, we will consider these three sensor-based manufacturing technologies. CPS is an infra-service that links the real world with diverse and complex processes and information, i.e., a computer-based component or system that is closely connected to the cyber world. In addition, CPS is a key technology in smart manufacturing that is closely linked to the cloud, IoT, and big data technologies in manufacturing. The core of the CPS is to simplify and systematize the relationship between the physical system and the control software from the design stage to secure a system for developing, operating, and managing reliability at a predictable level. We will be able to incorporate the main concept of CPS in this study. In other words, we think that it will be possible to create a newly added value such as productivity increase by implementing an intelligent smart factory, which is the final aim of this research [27,28]. IoT refers to a network of software and sensors connected with physical objects. This allows the user to collect information in real time. IoT enables the application of big data analytics by collecting and exchanging the data acquired from a smart sensor, which is regarded as a key technology for realizing smart manufacturing by implementing CPS and cloud manufacturing. IoT is required to be integrated from information-collection subunits such as sensors to the existing programmable logic controller (PLC) system in order to be applied to the manufacturing site. In addition, real-time manufacturing integration service (RTMIS) is required to build a service-oriented architecture (SOA)-based IoT platform to support real-time collection of manufacturing information and efficient operation and interoperability of various manufacturing data. That is, various infra technologies such as sensor and network technology, big data, cloud computing, artificial intelligence, and 3D printing must be combined in order for IoT to be possible. In this study, we will be able to build a new system by combining various technologies such as sensor, network, and cloud based on the concept of IoT. Third, smart sensors, which directly collect information on the manufacturing site, are key technologies for realizing smart manufacturing, along with IoT, in device-based hardware technology. Sensor technology is either developed independently or developed along with network or IoT technology for manufacturing applications. This is because it is very important to process the sensed values at the manufacturing site where a large amount of complicated noise is generated, and the correction and acquisition of data must be performed simultaneously. Recently, several kinds of physical wireless communication technologies have been developed and applied in the field, along with studies of wireless network architecture connecting sensors. These representative technologies are as follows. First is the transducer electronic data sheet (TEDS), a standardized manufacturing-related sensing data structure. Second is real-time Ethernet (RTE), a communication interface that supports real-time information exchange. Third is complex programmable logic device (CPLD), which is a smart sensor interface that can process different types of sensor data in real time and in parallel.

## 3. Design

### 3.1. Experimental Settings

As mentioned in the previous section, many studies on the vulcanization process have primarily investigated the characteristics of polymers with the chemical control of additives or the temperature control of vulcanization equipment. However, in this study, we analyzed the cause of defects in the vulcanization process by collecting big data using smart sensors and applying various analysis methods for the on-site proof of concept (PoC) of company-A, which manufactures shoe soles. The period of the PoC was 9 months from June 2017 to February 2018. We performed the PoC based on the data accumulated during this period. In addition, we have built an alarm function that can detect failure in real time by creating an outlier detection logic for the cause of the failure. For this study, we attached a total of 4 sensors (6 points) per 30 curing equipment (vulcanizers) of company-A. The sensor consists of three temperature sensors (internal temperature of the vulcanizer, platen temperature, and jacket temperature) and three pressure sensors (internal pressure of the vulcanizer, valve pressure, and shaping pressure). Each sensor measures 8 to 12 actual values per second, and the measured data are integrated into a Hadoop system in real time in text form via a PLC. Each set of log data is structured and stored in Hadoop supported by Hortonworks, which is a framework for the distributed storage and processing of big data. All of the IoT log collection servers that comprise this system are built in a cloud system supported by Amazon Web Services (AWS). Each component server constituting the system is composed as follows. First, we configure the log collecting Web server as an instance type (two CPUs with 4 GB memory each). Second, in the case of the broker instance that transmits information from the IoT device, we construct an instance system by additionally connecting a 100 GB hard disk drive (HDD) to enable stable data transmission. Finally, in the case of Hadoop, which stores all user information, a 1 TB HDD is additionally connected to accommodate instantaneously changing data. Generally, in the case of accounting information for financial institutions, especially card companies, a billion pieces of information come into the system every day. It accounts for about 4 terabytes per column in about 10 years, and there are more than 100 columns, consuming 40 terabytes per year. At this time, the configuration of Hadoop infrastructure is usually 8 servers, 10 slots of HDD, and 6 terabytes per slot, and a total storage space of 480 terabytes occurs. This is usually accommodated in three storage spaces, so an average of 160 terabytes can be used for such an infrastructure configuration. The actual infrastructure of the project, which is the source of this study, is as above. However, in actual manufacturing process, even though the number of control variables is small (6), about 14 sensor data per second comes in through more than 1,000 sensors. This is the amount that consumes 100 terabytes per year for a simple arithmetic expression. By using integrated data, we develop real-time predictive defect-detection models by using the statistical software packages SAS base 9.4 and SAS Enterprise Miner 13.1 (SAS Institute Inc., Cary, NC, USA), and each model can be transferred back to the PLC and used as an alarm system.

**Figure 2:**
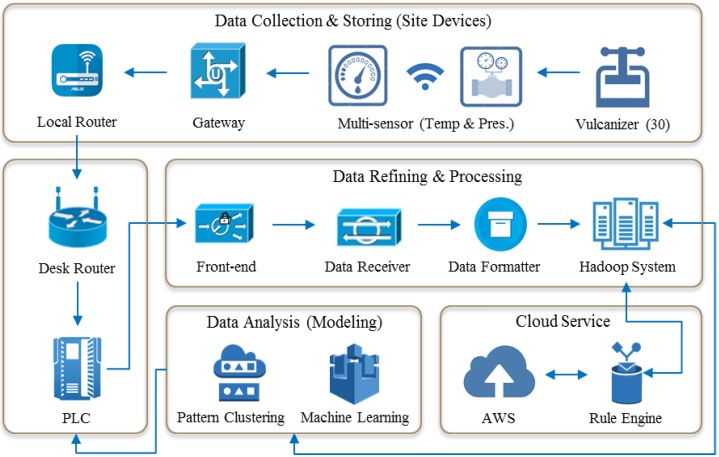
Sensor-based infrastructure.

Figure 2 shows a general overview of a real-time infrastructure system that collects, stores, analyzes, and generates models of measured data from each sensor. In detail, temperature and pressure information collected from the multi-sensor of each vulcanizer is wirelessly transferred to the PLC through a local gateway and router. At this point, all data are observed in 14 rows per second, and when transferred from local to desk, a time lag of 10–15 s usually occurs. However, the time lag is usually corrected during the data refinement phase. Depending on the manufacturing environment, distributed control systems (DCS) or manufacturing execution systems (MES) are often used in continuous steel or plastic processes. The above steps are usually referred to as the collection stage. Next, in the data refining step, we perform column formatting to fit the analysis logic. At this time, we remove the anomalies that do not reach the upper or lower level, set the time-lag correction, set the data security, and finally store the data in the Hadoop system. The formatted data are computed through pattern clustering logic, which is a type of machine learning methodology we have built through SAS. In addition, AWS updates the logic with new incoming data. This updated logic also provides the user with interpretation results for new outcomes (early warning signs). This infrastructure has been adopted and used by many organizations and manufacturers through several similar projects. For example, it is widely used not only in plastic-film and tire manufacturing companies, but also in home IoT service providers and for sensing customer movement patterns in shopping malls. We acknowledge that the infrastructure we use is a very common form of a big data solution and that this infrastructure is utilized in many different industrial domains. However, application to different industries does not require completely different mechanisms. In other words, data accumulation, data refinement, and updating of analytical algorithms using sensors are limited to industries with very similar patterns. However, the process of modeling can vary significantly from one industrial domain to another, and it is easily deduced that the analytical methodology can be very different. Therefore, what we emphasize in this study is to focus more attention on the differences in the modeling methodology than on the explanation of this general and typical big data infrastructure. In particular, the analytical methodology is significantly different from that used in the past, and it has not been practically applied so far.

Each of the four sensors attached to all 30 vulcanizers simultaneously measures both temperature and pressure and transmits data to the PLC. The platen temperature and shaping pressure are measured using the same method. Company-A vulcanizes SBR raw material for approximately 12 min on average. The jacket that presses up and down in the vulcanizer is a direct source of heat, with which the platen forms a shape. The internal temperature refers to the temperature of the internal space of the jar in which the SBR is formed. In the case of a pressure sensor, we also measure the internal pressure through a sensor that measures the internal temperature. In addition, we take the reading of the pressure control instrument (PCI) and measure the shaping pressure. At this time, if the internal pressure is the actual value of the pressure controlled by the user, the PCI pressure is the value measured by the vulcanizer itself, which is slightly different from the actual pressure value. Since the shaping pressure is a measure of the pressure directly applied to the SBR, we can define it as a value that has the most substantial effect on the final product. In this manner, we installed four multi-sensors that measure temperature and pressure at six points in all 30 vulcanizers. In addition, we measured the temperature and pressure at the time when the vulcanizer reached the maximum temperature and maximum pressure on average. These values are listed in Table 3 as the mean values and the 2.5 standard deviation values 8 min after the start of the vulcanization. Based on the measured temperatures, we found that the internal temperature was higher and the standard deviation was lower than the temperature of the jacket. We attributed this result to the time difference between the measurement of internal temperature, which was performed in the gaseous state, and the measurement of jacket temperature, which was performed in the solid state. In other words, assuming that the same temperature is maintained at a certain point in time, we can state that the convection in air is somewhat slower than the heat transfer by conduction between the objects. Based on the measured pressures, the PCI pressure is somewhat lower than the internal pressure, indicating that there is a time lag between the actual and measured values. In addition, the shaping pressure is higher than the other two pressure indicators, indicating that the effect is very direct.

### 3.2. Pre-Processing

Vulcanized rubber materials are usually tested for thermal properties to determine if the heat has been homogeneously applied. This is a type of test to identify defective products with shrinkage and cracking due to variations in in rubber elasticity. At this time, the specific heat of the rubber is measured, which is the calories (Cal/g. °C) required to increase the temperature of the object by 1 °C. For SBR, the normal static specific heat is 1.88–2.09. In addition, the thermal expansion of rubber is a very important indicator. That is, since rubber has some characteristics of liquids and is sensitive to heat, its thermal expansion is measured to determine whether it is normal. In the case of SBR, it can be said that the condition is normal if the thermal expansion coefficient is between 1.3 and 2.2. Finally, one of the most important items in defect inspection is the test of vulcanization rate. The optimum vulcanization rate varies depending on the use of the product, but in the case of SBR for shoe soles, the optimum vulcanization rate is determined by the vulcanization shrinkage ratio, which is a proportional function of thermal expansion rate, and the specific heat capacity of rubber is between 1.5 and 2.5. The main property test of these synthetic rubber products is based on a system called “foolproof system.” Company-A conducts inspections of finished products for each product code produced daily through statistically significant sampling methods. The items to be computerized are classified into 11 types according to the type and size of shoes such as formal shoes, sneakers, and slippers. Company-A collects the specific heat value through the thermal property test and manually determines the vulcanization coefficient through the optimum vulcanization degree in the form of a spreadsheet in its own database. Specifically, the test is performed 12 times every 2 h by randomly sampling 11 types of sole products from 30 vulcanizers operating 24 h a day. As mentioned above, the specific heat is measured from the amount of heat required to raise the product temperature by 1 °C, and the vulcanization coefficient is derived by calculating the rubber capacity and measuring the thermal expansion rate. Therefore, the data in this study include six types of curing-process sensor data and two types of manual inspection data (specific heat and sulfur coefficient). For generating the master data table, we performed an inner join of the inspection data table containing both specific heat (SH) and sulfur coefficient (SC) with the vulcanization data table that indicates the product uniformity by process time. Moreover, we distinguished the outliers of vulcanization sensing data, which usually do not vary significantly, to facilitate the analysis. For this purpose, we identified the largest (MAX) and smallest (min) values for a specific product, a specific time zone, and a specific rounding process, in addition to the measured raw value. We eventually parameterized it in the master data. In order to examine the difference between the user setting value and the actual value, we show the interval for a value that is 1.5 standard deviations from the average and treat it as a dummy variable.

### 3.3. Methodology

We analyzed the vulcanization process using two methods. The first is a general statistical analysis method, in which we set input and target variables first and then identify the significance of the two variables. We used regression and decision trees (DTs) as the analytical methodology. In addition, we intend to increase the robustness of the statistical significance by using machine learning. We used gradient boosting (GB), random forests (RF), and AOV16 provided by SAS e-Miner as the ML methodology. Gradient boosting is machine learning technique for solving regression or classification issues. It creates a prediction model and is mainly used to strengthen weak methods, typically decision trees. Random forests is an ensemble learning method for classification, regression, and other tasks, and it operates by constructing a multitude of decision trees in the training time and outputting the class that is the mode of the classes or mean prediction (regression) of the individual trees. AOV16 is one of the methodologies provided by SAS e-Miner. When we perform a variable selection for interval-scaled (or continuous) input, we refer to the R-square value of the target and the binned version of the input variable. At this time, the binned variable is a categorical variable created by the variable selection node. The levels of this categorical variable are the bins. In SAS Enterprise Miner, this binned variable is referred to as an AOV16 variable. The number of levels or categories of the binned variable (AOV16) is at most 16, corresponding to 16 intervals that are equal in width. 

The second method is pattern clustering. We believed that the pattern of the time flow of variables can be confirmed through the clustering of all processes. It can be implemented through the similarity procedure of SAS e-Miner. The above-mentioned first-round analysis must be preceded and accompanied by the specific heat and sulfur coefficient tests, which are tests of chemical properties. This also means that automation is difficult. Furthermore, in the case of the test of physical properties, sampling is performed at intervals of 2 h. This means that the vulcanization data measured in chronological order have a considerable time difference from the chemical test data, which are calculated manually after the end of the process. To overcome this problem, we devised a defect-detection model based on pattern clustering. In other words, we did not use the target variable, or physical test data; rather, we used only the abnormal flow of the input variable. Through this approach, we have determined the causative variables and the exact timing of abnormal phenomena. Table 4 summarizes the analysis method.

## 4. Experimental Evaluation

### 4.1. Result of 1st-Round Analysis

The significant responses of the input variables to the outliers of the target variables were in the following order: Shaping pressure, internal pressure, jacket temperature, platen temperature, internal temperature, and PCI pressure. This is the result of verifying the statistical significance among regression, decision trees, and three machine learning algorithms (GB, RF, and AVO16) in terms of both input and target variables. As summarized in Table 5 we determined significance as *p*-values both under 0.05 and under 0.1. We first performed the general statistical analysis (regression and DT) and found that the variation of sulfur coefficient was larger than that of specific heat. Consequently, the significance was also clearer. Therefore, we designated only the sulfur coefficient as the target variable in the machine learning series analysis. The results of the first analysis are summarized as follows. First, pressure has a greater possibility than temperature to cause an abnormality. Among the six input variables, the ones showing the highest significant reactions are shaping and internal pressure. The PCI pressure is the least significant because of the time lag with the actual value of the PCI pressure. Therefore, there is a problem in using this as a criterion for determining the significance of an actual product defect. Second, shaping pressure and jacket temperature, which have more direct effects on the product than internal pressure and temperature, are more significant for both *p*-value conditions. Third, in terms of methodology, the decision tree series shows more significance than the regression series. In particular, in the case of the machine learning series, the random forests based on decision trees show a greater significance for the target variable sulfur coefficient than the regression-based AOV16. For reference, in the analysis of gradient boosting (0.5 and 0.3) and random forests (90 trs, 60 trs), we made a difference in index adjustment. This is generally intended to clarify the significance through buffering in bootstrapping, variable amplification, or tree division. The lower the index value is, the less stringent will be the criteria applied.

### 4.2. Results of 2nd-Round Analysis

As mentioned earlier, the 1st-round analysis should be accompanied by the inspection of specific heat and sulfur coefficient. This means that automation is impossible. In addition, there is a problem that the target variable cannot be interlocked with the time axis of the input variable, which is the time series data. To overcome this issue, we devised a defect-detection model through pattern similarity analysis. In other words, we made a model based only on sensing data by classifying clusters with similar flow based on the change pattern of the input variable without depending on the target variable [29].

Clustering methods require a more precise definition of “similarity” (“closeness”, “proximity”) of observations and clusters. When the grouping is based on variables, it is natural to employ the familiar concept of distance. The Euclidean distance between the two points is the hypotenuse of the triangle ABC when three angles of triangle are A, B and C respectively.

$$D_{2}\left(i,j\right)=A^{2}+B^{2}={\left(X_{1i}-X_{1j}\right)}^{2}+{\left(X_{2i}-X_{2j}\right)}^{2}$$

The measure of distance depends on the units in which *X*_1_ and *X*_2_ are measured, and is influenced by whichever variable takes numerically larger values. For this reason, the variables are often standardized so that they have mean 0 and variance 1 before cluster analysis is applied. Alternatively, weights *w*_1_, *w*_2_, …, *w_k_* reflecting the importance of the variables could be used and a weighted measure of distance calculated.

$$D\left(i,j\right)=\sqrt{w_{1}{\left(X_{1i}-X_{1j}\right)}^{2}+w_{2}{\left(X_{2i}-X_{2j}\right)}^{2}+\ldots +w_{k}{\left(X_{ki}-X_{kj}\right)}^{2}}$$

The process of pattern similarity analysis (statistically, k-means clustering with Euclidean distance and procedure similarity in the function name of SAS) is as follows. First, we identified all product flows from the data of the six sensors over a 12-min process in which one sole product was produced. The method to distinguish typical variations is the use of visualization tools such as a dendrogram, a constellation chart, and a parallel-coordinate plot provided by SAS e-Miner, and we can also obtain each value of the statistical significance test for visualization. Second, each cluster is automatically classified into a few clusters according to the shape of the data flow. We extract the non-mainstream clusters, which are different from the mainstream clusters. Third, we trace which vulcanizers produced these abnormal clusters and at what time they occurred. Fourth, we reaffirm that the object produced by the vulcanizer at that time is actually defective. To do this, we analyze the specific sensor value and output information of the vulcanizer. Finally, once the problem with the reassessed sensor value becomes clear, we create the alarm system and the revision model so that the user can adjust the setting value to correct it. We built this model into the PLC system for the user.

**Figure 3:**
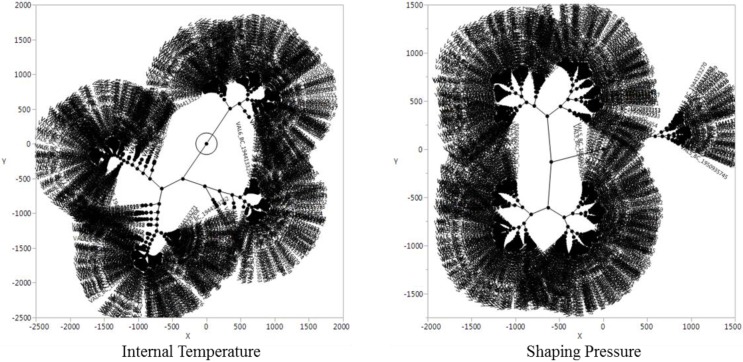
Constellation chart.

Figure 3 shows a constellation chart of internal temperature and shaping pressure. As can be seen from the chart, the distances among the clusters from the two sensors are distinctly different. In the case of internal temperature, the distance between the clusters is based on a single and large point, while the shaping pressure forms another minor cluster separately from the mainstream cluster.

**Figure 4:**
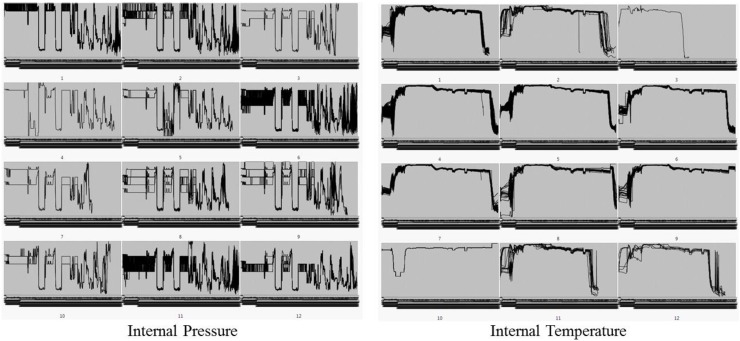
Parallel-coordinate plots from the pattern similarity cluster.

Figure 4 shows parallel-coordinate plots that visually classify the pattern similarity by overlapping all sensing data of internal pressure and internal temperature. We could not find any significant difference in the other sensing data according to the significance test provided by the cluster analysis. However, in the case of shaping pressure, we have found non-mainstream clusters of outliers.

**Figure 5:**
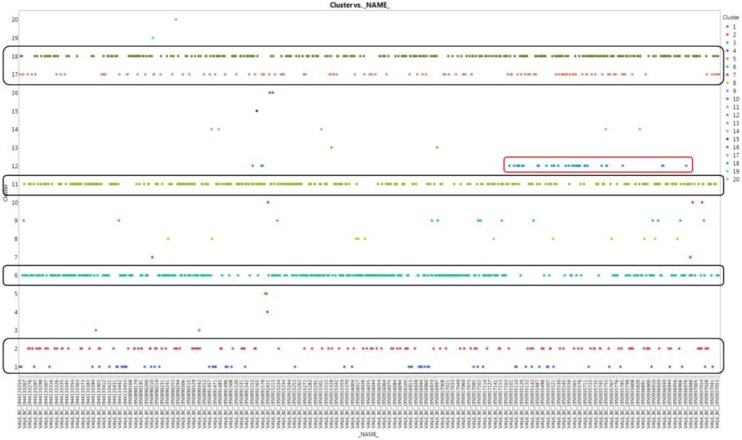
Cluster type by time stream for shaping pressure.

In Figure 5, for clusters No. 1, 2, 6, 11, 17, and 18, a typical stream in each specification is formed, and the remaining clusters appear to be bundles of random values, rather than specific streams. However, in the case of cluster No. 12, the flow from the middle to the end seems to be a constant flow, rather than a random flow, and we judged it as an abnormal symptom.

**Figure 6:**
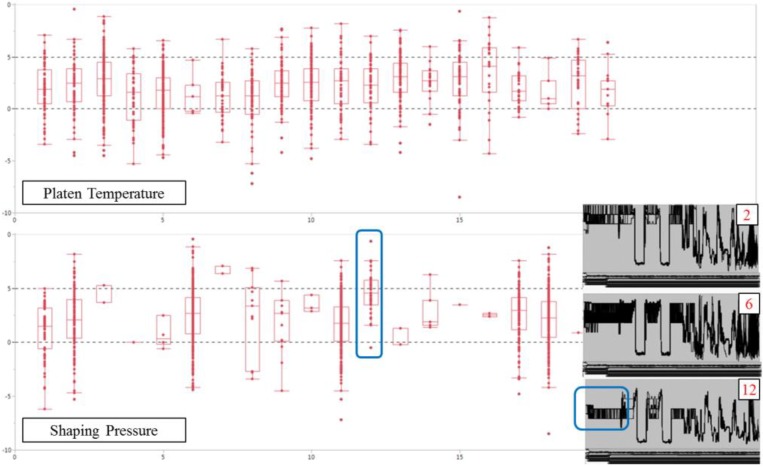
Box plot from the clusters of platen temperature and shaping pressure.

We made box plots for each cluster of shaping pressure, as shown in Figure 6. A box plot of the platen temperature cluster with low significance for variability is also presented for comparison. We find that the median values of clusters No. 1, 2, 6, 11, 17, and 18 are located at a significant level in the case of shaping pressure, like cluster type according to time order. However, cluster No. 12 has a significantly different median value from the other clusters. Likewise, as the parallel-coordinate plots show, the early shaping pressure of cluster No. 12 begins somewhat lower than that of the other significant clusters.

**Figure 7:**
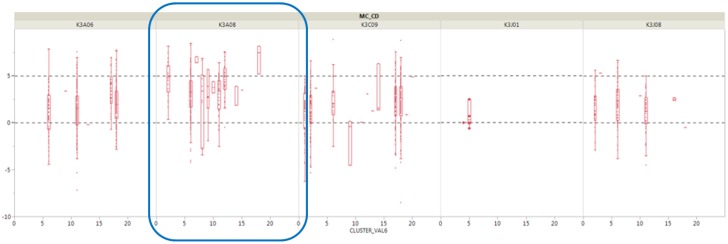
Box plot from the clusters of the specific vulcanizer.

To find more specific causes, we sought where cluster No. 12 of shaping pressure was derived from the 30 vulcanizers. We also made box plots by crossing existing clusters by each vulcanizer, as shown in Figure 7. The result shows that the median value of a vulcanizer (curing device No. K3A08) is higher than the average for all vulcanizers.

**Figure 8:**
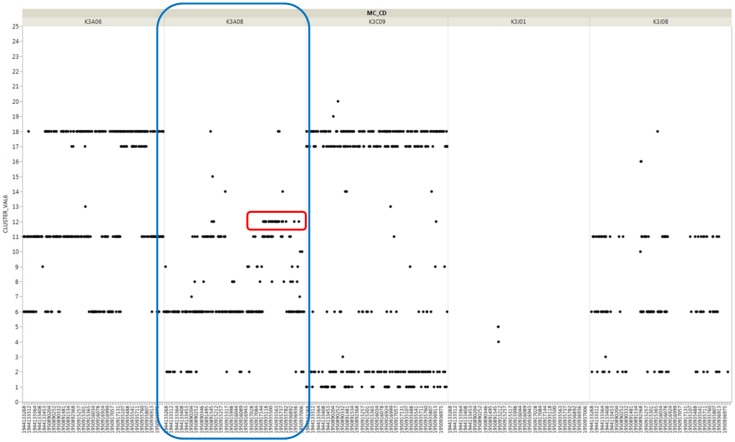
Cluster type of vulcanizer K3A08 by time stream.

In addition, we examined the cluster type according to the time-series flow of vulcanizer K3A08, as shown in Figure 8. We found that cluster 12, which was judged to be problematic in pattern similarity, appeared mainly in vulcanizer K3A08. In addition, we can see that there is sufficient temporal significance as the anomaly starts from the end of 2017 as a result of pattern similarity.

## 5. Conclusions

Previous studies on most vulcanization processes have investigated the properties of polymers through the chemical control of additives and temperature control of vulcanization equipment. However, such research has not been conducted at the actual site; rather, it has been conducted by controlling the physical properties by using machines in the laboratory. In this study, we have attempted to use various analysis methods ranging from conventional statistical analysis to ML and pattern analysis using PLC sensor data in an actual manufacturing process. Furthermore, we created an alarm model that identifies the cause of failure in advance. In addition, we have proved that a highly efficient defect pre-detection system can be created with only sensor data by using the existing experimental method of creating target variables through the inspection of chemical and physical properties. We think many manufacturers are having a problem in not conducting the defect inspection in real time. We have found that this is due to the fact that the testing of the final product must be carried out in the laboratory. We also know that, in this case, there is a time lag between the manufacturing process and the test, which makes it difficult to identify the exact cause. Therefore, we are confident that the methodology of this study, which excludes dependent variables and identifies the causes with only independent variables, will be widely used in many future manufacturing analyses.

In summary, the reason why our research is academically superior is as follows. First, we apply not only general statistical analysis, but also ML analysis. We have established stronger support for the significance of existing causal relationships through ML methodologies such as GB, RF, and AOV16. Second, we demonstrate that we can show the significance only through explanatory variables without using dependent variables. In fact, many data analysis studies failed to achieve a reliable symmetry of input and target variables, which has caused a loss of confidence in the research. We can recreate the results of the causal relationship from the existing analysis through the pattern similarity cluster for the input variables so that we can demonstrate of the core idea of our research. Third, we have established a new academic field by combining the field of synthetic rubber materials, which was mainly limited to polymer research, with sensors and big data infrastructure technology. As mentioned earlier, previous research on synthetic rubber can be divided into two broad categories: The reduction of the defective ratio through vulcanizer control and the development of new materials through the mixing of additives. In contrast, we have made a new attempt to combine various methodologies, sensors, and infrastructure technologies. Our research also makes a considerable practical contribution. First, if the precautionary model works without the inspection of chemical properties, it can shorten the time and effort required for the conventional chemical test. Second, unlike existing systems, vast quantities of data are stored on the Hadoop system and in the cloud; therefore, there is no need to purchase DBMSs or manage data. Moreover, since the model we built is a real-time model with logic embedded in the PLC, it can be updated at any time. That is, the risk of additional cost burden on the user’s side is considerably small. Third, we have gained a broader range of reference data in the progressive aspects of emerging big data systems for service-oriented manufacturing, such as CPS, IIoT, and RTMIS. The performance of a technology cannot be guaranteed without clear proof obtained from application in the industry. That is, empirical evidence, including productivity improvements, resource savings, or the improvement of accounting standards, is very important. This study conducted an empirical study on big data technology, which has proven academic excellence, by using a big data system and machine learning methodology. In addition, it is very important to apply the spread of a similar industrial domain in the future through securing such an important reference. In this study, we have left enough room to analyze not only the vulcanization process but also the molding process, which can result in the accumulation of more sensor data because of the process characteristics.

## Figures and Tables

**Table 1:** Types of synthetic rubber.

Type	Feature	Products
SBR (styrene butadiene rubber)	Stress-cracking resistance, abrasion resistance, aging resistance, and thermal resistance	Tires, shoe soles, rubber hoses, wire clothing, floor mats, adhesive tapes, etc.
BR (polybutadiene rubber)	Excellent elasticity, cold resistance, and abrasion resistance	Airplane tires, shoe soles, vibration-proof rubber, rubber rolls, etc.
CR (chloroprene rubber)	Chemical resistance, ozone resistance, aging resistance, and thermal resistance	Wire clothing, conveyor belts, waterproof rubber, window-ledge rubber, etc.
NBR (nitrile butadiene rubber)	Oil resistance, abrasion resistance, and aging resistance	Oil hoses, print rolls, weaving top rolls, oil caps, conveyor belts, etc.
IRR (isobutylene isoprene rubber)	Gas resistance and ozone resistance	Tire tubes, curing bags, wire clothing, steam hoses, conveyor belts, etc.
EPDM (ethylene propylene terpolymer)	Electromagnetic resistance, aging resistance, and ozone resistance	Wire clothing, steam hoses, water streams, conveyor belts, etc.

**Table 2:** Summary of recent studies on vulcanization.

Category of Research Area	Description	Representative Literature
Adjust the conditions of the curing system	A research method that measures the performance of the product with different curing temperatures or time conditions of the curing system	Rabiei and Shojaei [8]; Garraza, et al. [9]
Measure the effect of vulcanization accelerators	A study comparing existing results for the performance resulting from adjusting the ratio between the rubber material and accelerator components, adding new accelerators, or changing external environmental conditions	Mansilla, et al. [10]; Zhong, et al. [11]; Mansilla, et al. [12]
Change the cross-link formation of polymer chains	A more chemically based sequencing study to prevent defects or to produce improved synthetic rubber by changing the cross-linking system between the polymer chains of SBR and sulfur	Chen, et al. [13]; Boonkerd, et al. [14]; Shao, et al. [15]
Utilize sensor devices for time-based data collection	A study for collecting well-ordered time series data to fine-tune the effects of different situations and to determine the exact cause of tiny and irregular variations	Ponnamma, et al. [16]; Chen, et al. [17]; Ma, et al. [18]
Prevent environmental pollution	Research emphasizing pollution prevention and the improvement of environment-friendliness of the vulcanization process of synthetic rubber	Dobrotă, et al. [19]; Sienkiewicz, et al. [20]
Introduce a new material with various mixing conditions	A study introducing advanced rubber products and related compounds, as well as their special curing methods	Hernández, et al. [21]; Lin, et al. [22]
Use a novel manufacturing technology	A study on the development of sustainable applications for improving the manufacturing environment and the practical performance of the synthetic rubber industry	Xiang, et al. [23]; Basterra-Beroiz, et al. [24]

**Table 3:** Values measured using vulcanizer sensors.

Measurement (2 Types)	Sensor (6 Points, 4 Sensors)	Value (Measured at 8 min, Average ± 2.5 Standard Deviation)
Temperature	Internal temp.	183 °C ± 5.4 °C
	Jacket temp.	171 °C ± 8.6 °C
	Platen temp.	165 °C ± 6.4 °C
Pressure	Internal pres.	23.7 kgf/cm^2^ (23.2 kPa) ± 2.4 kgf/cm^2^
	PCI pres.	23.2 kgf/cm^2^ (22.7 kPa) ± 2.1 kgf/cm^2^
	Shaping pres.	24.1 kgf/cm^2^ (23.6 kPa) ± 2.8 kgf/cm^2^

**Table 4:** Analysis methodology of vulcanization process.

Sequence	Methodology	Description
1	Exploratory factor analysis (specific sole type)	Analyze the volatility of the specific sole type (SBR for climbing shoes, which uses relatively more raw materials) in order to increase the effectiveness of the analysis
2	Generation of input & target variables	Create new target variables (linearized discrete variables SH and SC) and derive input variables (MAX and min values generated by a unit of the product in a minute)
3	1st-round analysis (causal equation)	Identify associations of overall variables (Regression and DT) and select the importance of variables (GB, RF, AOV16)
4	2nd-round analysis (pattern cluster)	Perform cluster analysis on the selected causal factors

**Table 5:** Result of 1st-round analysis.

Analysis Type	Temperature			Pressure		
	In-Temp.	Jacket	Platen	In-Pres.	Shaping	PCI
SH (DT)	2415.36 *	521.44 *	2.2254 *	22.54 **	31.85 **	5.87
SH (Reg.)	132.58	24.36	0.2148	2.11	5.35 *	1.09
SC (DT)	2843.11 *	668.37 **	5.2218 **	31.73 **	29.25 **	16.39 *
SC (Reg.)	95.14	21.41	0.5250	5.33 *	4.92 *	4.84
GB (≥0.5)	43.84	10.25	0.8547	42.77	68.36 *	10.60
GB (≥0.3)	32.88	87.91 *	0.3517	67.13 *	82.64 **	9.77
RF (≥90 tree)	2.1425 *	4.8511 *	2.2545 *	53.21 *	81.98 **	12.83
RF (≥60 tree)	2.0218 *	5.2145 **	1.9218 *	121.55 **	116.28 **	48.36 *
AOV16	182.12	177.61 *	0.5844	5.19 *	5.02 *	1.10
# of Significance	4	6+	4+	7+	8+	2
Rank	5	3	4	2	1	6

(*t*-value, *: *p* ≤ 0.1, **: *p* ≤ 0.05).
